# Investigation of a monophasic *Salmonella* Typhimurium outbreak linked to chocolate products as part of wider international outbreak: A matched case–control study, Ireland, 2022

**DOI:** 10.1002/puh2.116

**Published:** 2023-11-20

**Authors:** Charlotte Salgaard Nielsen, Patricia Garvey, Martin Cormican, Niall DeLappe, Mary Lenahan, Orla Moore, Kay Casey, Suzanne Cotter, Sarah Gee, Benjamin Aniugbo, Gerard Meagher, Elaine Brabazon, Keith Ian Quintyne, Anthony Breslin, RoseMary McBride, Eimear Burke, Margaret White, Marie Casey, Leah Evans, Catherine Conlon, Annette Dillon, Regina Kiernan, Donna Kilmartin, Cale Lawlor, Ruth McDermott, Paul McKeown, Outbreak Control Team

**Affiliations:** ^1^ Health Service Executive (HSE)—Health Protection Surveillance Centre (HPSC) Dublin Ireland; ^2^ ECDC Fellowship Programme Field Epidemiology Path (EPIET) European Centre for Disease Prevention and Control (ECDC) Stockholm Sweden; ^3^ National *Salmonella* *Shigella* & Listeria Reference Laboratory Galway Ireland; ^4^ Food Safety Authority of Ireland Dublin Ireland; ^5^ Environmental Health Service Health Service Executive Dublin Ireland; ^6^ Department of Public Health Health Service Executive—Midlands Dublin Ireland; ^7^ Department of Public Health Health Service Executive—North‐East Dublin Ireland; ^8^ Department of Public Health Health Service Executive—North‐West Dublin Ireland; ^9^ Department of Public Health Health Service Executive—South‐East Dublin Ireland; ^10^ Department of Public Health Health Service Executive—Mid‐West Dublin Ireland; ^11^ Department of Public Health Health Service Executive—South Dublin Ireland; ^12^ Department of Public Health Health Service Executive—West Dublin Ireland; ^13^ Department of Public Health Health Service Executive—East Dublin Ireland

**Keywords:** chocolate, foodborne disease, outbreak, *Salmonella* Typhimurium

## Abstract

**Background:**

In March 2022, an outbreak investigation was initiated in Ireland after identifying a cluster of monophasic *Salmonella* Typhimurium cases, affecting primarily small children. Microbiological investigations revealed that the cluster was part of a wider international outbreak.

**Methods:**

A total of 18 Irish outbreak cases were identified. We undertook a matched case–control study using the case–case method to determine if exposure to the implicated products was associated with illness.

**Results:**

In univariable analysis, the highest odds of disease due to monophasic *S*. Typhimurium versus other gastrointestinal disease were obtained for a chocolate Product A of Brand A [matched odds ratio (mOR) = 7.77, 95% confidence intervals (CI): 0.89–67.20]. When grouping the implicated products in a composite variable, the odds of disease due to monophasic *S*. Typhimurium versus other gastrointestinal disease were 10.5 times higher with a consumption of at least one of the implicated products [mOR = 10.50, 95% CI: 1.24–88.60, *p* = 0.031].

**Conclusion:**

This analytical study supported the internationally generated hypothesis which led to the implementation of control measures. Owing to the high levels of chocolate purchasing with Easter approaching, early outbreak identification and involvement in the internationally coordinated investigation was essential to an efficient response and to minimise the risk of further harm to a particularly vulnerable population group.

## Graphical abstract

Graphical Abstract:

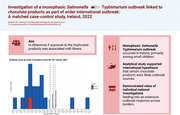

## INTRODUCTION

The reported incidence rate of salmonellosis in Ireland is relatively low compared to other parts of the European Union (EU) [1], with 173–414 cases reported per annum in the period 2017–2021 [2, 3]. On 24 March 2022, a cluster of seven cases of monophasic *Salmonella enterica* subsp. *enterica* serovar Typhimurium (i.e. monophasic *Salmonella* Typhimurium) of sequence type (ST) 34 was identified in Ireland. Cases presented with an unusual epidemiological profile, with predominantly female children below 10 years of age affected. Microbiological investigations revealed that the cluster was part of a wider international outbreak, for which an EpiPulse alert had been issued by the United Kingdom (UK) on 17 February 2022. Responses to this alert, by a number of countries, indicated an extensive outbreak.

The outbreak evolved rapidly, with 150 reported cases in nine EU and European Economic Area (EEA) countries and the UK by 8 April 2022 [4]. This resulted in extensive collaboration among the affected EU Member States, the European Centre for Disease Prevention and Control (ECDC) and the European Food Safety Authority in order to investigate the outbreak source and coordinate the response. There was close coordination throughout with the UK authorities.

International epidemiological investigations based on case interviews indicated that a high proportion of cases had been exposed to specific chocolate products of Brand A produced by Company A. Microbiological investigations identified two distinct clusters of *S*. Typhimurium, with most cases belonging to the first. Following official controls at a Belgian processing plant, the hypothesis was strengthened by positive *Salmonella* sampling in the production environment of Brand A, of which a number of isolates matched the two outbreak strains. In Ireland, the first products were recalled on 2 April 2022, and the withdrawal of authorisation for production at the implicated plant and product recalls was extended to all batches of Brand A by 8 April 2022 [5].

Hypothesis‐generating interviews of the confirmed cases in Ireland supported the internationally generated hypothesis that chocolate products of Brand A were the likely outbreak vehicles. We conducted a matched case–control study using the case–case method to determine if exposure to the implicated products was associated with illness.

## METHODS

### Study design, participants and sampling

A matched case–control study using the case–case method was conducted between March and April 2022. Any case meeting the international case definition as defined by the ECDC [5] and notified on or after 7 March 2022 as part of outbreak cluster 1 were included in the study. This cut‐off date was chosen owing to the proximity to the start of the data collection in order to limit recall bias among the study participants. Confirmed cluster 1 cases were matched by age and sex with cases of other gastrointestinal disease (1:3 ratio) notified within the same two‐week period as their matched case. Cases and controls were interviewed by telephone by Departments of Public Health using a questionnaire designed to collect information on demographics, clinical presentation (cases only) and selected food exposures, including a comprehensive selection of 21 chocolate products of Brand A produced by Company A, five chocolate products of Brand B produced by Company A and 13 alternative child‐friendly snack foods.

It was estimated that a sample size of nine cases and 27 controls would detect an association with an odds ratio (OR) of 10 at a 5% significance level with 80% power, in a matched case–control study with a case–control ratio of 1:3, and assuming 20% consumption of the implicated products among the controls.

**Impacts**

- A monophasic *S*. Typhimurium outbreak occurred in Ireland, primarily among small children, and was soon after identification linked to a wider international outbreak.
- Irish analytical epidemiological investigations supported the internationally generated hypothesis that certain chocolate products were the likely sources of the outbreak.
- This outbreak investigation demonstrated the value and importance of individual national investigations feeding into the coordination of an extensive outbreak response across borders to alleviate the risk of harm to a particularly vulnerable population group.

### Setting and outbreak detection

Ireland is an island off the coast of North‐Western Europe, with a population of 5,149,139 in 2022 [6]. Although salmonellosis is a common infection of the gastrointestinal tract caused by the bacterium *Salmonella*, the number of human cases of salmonellosis has progressively decreased in Ireland since 2000. According to the latest surveillance data from 2021, the national notification rate of salmonellosis was 3.5 per 100,000 population in comparison to an EU notification rate of 16.7 per 100,000 population [7]. *Salmonella* serotype Typhimurium and monophasic Typhimurium are the most common serotypes among cases acquired in Ireland followed by *Salmonella* serotype Enteritidis [8].

In Ireland, salmonellosis is a notifiable disease under Irish Infectious Disease Regulations 1981 and subsequent amendments. Clinicians and directors of clinical microbiological laboratories are required to notify cases to Public Health authorities. Outbreaks may be detected through epidemiological links between cases or, as in this case, based on the similarity of deoxyribonucleic acid (DNA) sequences between isolates submitted to the National *Salmonella*, *Shigella* & *Listeria* Reference Laboratory (NSSLRL) by clinical laboratories.

For this outbreak, a confirmed case was defined as a person living in Ireland with laboratory confirmed infection with monophasic *S*. Typhimurium, with a sequence consistent with the wider international outbreak [5]. Cases were identified through the mandatory notification system in combination with sequencing results reported by the NSSLRL.

### Laboratory methods

To undertake whole genome sequencing (WGS), DNA was extracted from isolates using the EZ1® DNA Tissue kit (Qiagen Gmbh). Sequencing libraries were prepared with Nextera DNA Flex library prep kit (Illumina), and WGS was performed on the Illumina MiSeq platform with 2 × 300 bp paired‐end reads (MiSeq reagent kit, version 3). BioNumerics 8.1 was used to predict serotype and determine the core genome multi‐locus sequence typing (cgMLST) by gene‐by‐gene comparisons for all genes defined as core genes. Isolates were considered indistinguishable when sequences were identical for all alleles defined as part of the core genome. Irish cases were considered part of the outbreak if they met the laboratory criteria for cluster 1 or 2 in the international outbreak: a monophasic *S*. Typhimurium ST34 isolate clustering with any of the representative outbreak isolates by the national cgMLST pipeline within five allelic differences.

### Statistical methods

Frequencies and percentages were calculated for outbreak case characteristics for all outbreak cases and by cluster. Conditional logistic regression was used to calculate matched OR (mOR) and 95% confidence intervals (CI). Forward stepwise selection of variables added in the order of the lowest *p*‐value was followed in a multivariable analysis by considering those exposure variables with a low *p*‐value (< 0.2) and OR > 1 in the univariable analysis. All analyses were carried out using R (version 4.1.3, R Core Team, 2021).

### Ethics approval

The analytical study was exempt from ethical committee approval, as such a study falls within routine operational practices conducted in the interest of public health. The legal basis for undertaking this work lies under the statutory Medical Officer of Health functions in Ireland outlined under Infectious Diseases Regulations 1981 – Regulation 11.

## RESULTS

### Descriptive epidemiology

**FIGURE 1:**
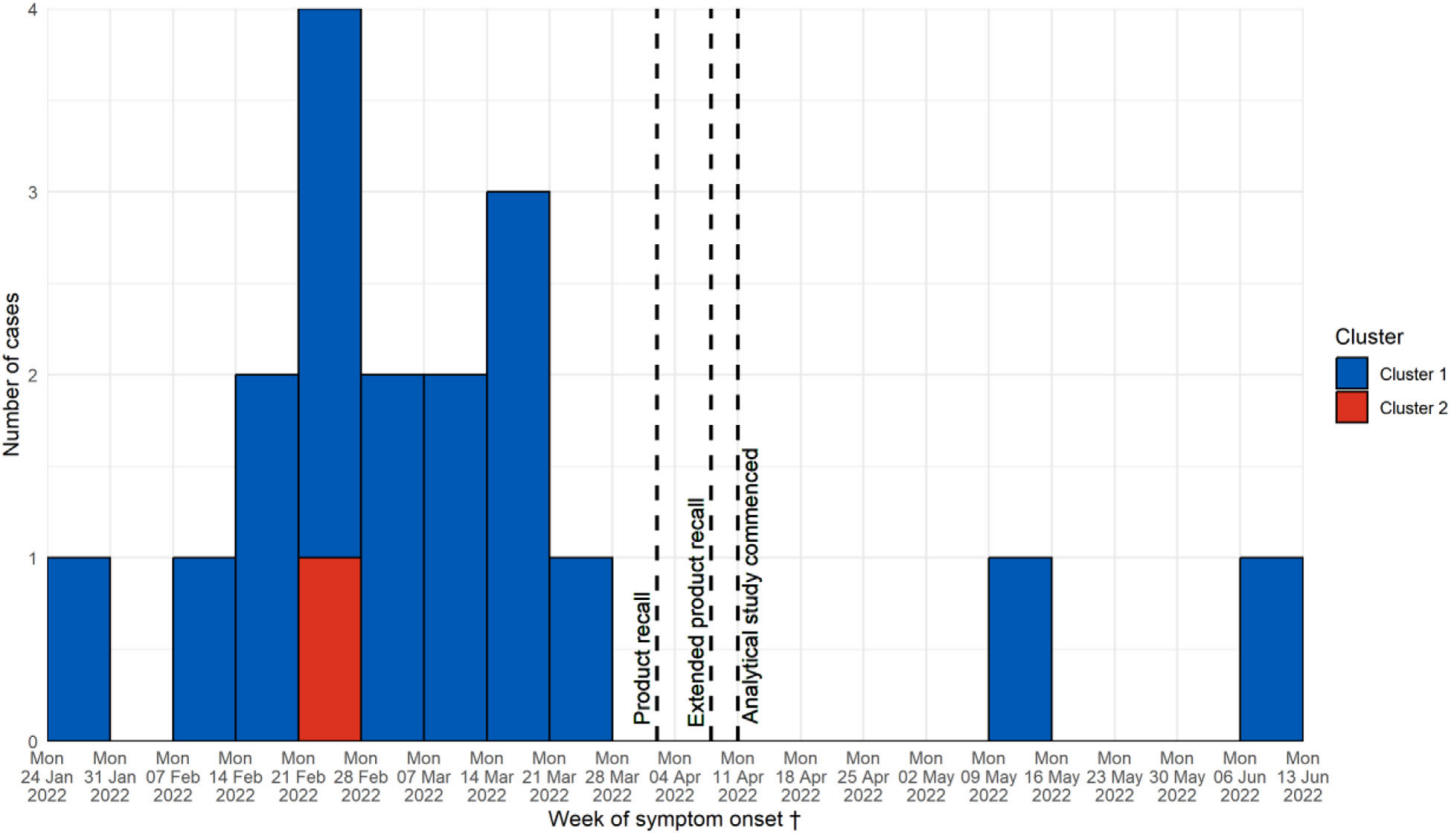
Monophasic *Salmonella* Typhimurium outbreak cases by week of symptom onset, Ireland, 2022 (*N* = 18). ^†^Date of diagnosis was used a proxy for two cases without reported symptom onset.

By 4 April 2022, 15 cases in Ireland had isolates indistinguishable on cgMLST from the main cgMLST pattern associated with outbreak cluster 1, and one case was indistinguishable on cgMLST from outbreak cluster 2. The shape of the epidemic curve was consistent with a continuous source outbreak (Figure 1). Two additional cases belonging to cluster 1 were notified on 25 May and 13 June 2022, after the analytical study had been concluded.

The first confirmed Irish case linked to the outbreak was notified on 8 February 2022 with symptom onset on 29 January 2022. Cluster 1 cases had symptom onset, primarily defined by diarrhoea, ranging from 29 January to 9 June 2022. One additional case with symptom onset on 25 February 2022 was part of cluster 2.

As presented in Table 1, the majority of cases were female (81%) and less than 10 years of age (81%). Of those reporting symptoms, 14 (87.5%) developed diarrhoea, and 7 (43.5%) developed bloody diarrhoea. Four cases (25%) were hospitalised.

**TABLE 1:** Characteristics of outbreak cases (*N* = 18), monophasic *Salmonella* Typhimurium outbreak, Ireland, 2022.

		Cluster 1 (*n* = 17)		Cluster 2 (*n* = 1)		Total (*N* = 18)	
Characteristics		*n*	%	*n*	%	*n*	%
Age (years)	0–4	9	52.9	0	0	9	50.0
	5–9	5	29.4	1	100.0	6	33.3
	≥10	3	17.6	0	0	3	16.7
Sex	Female	14	82.4	1	100.0	15	83.3
	Male	3	17.6	0	0	3	16.7
Symptoms^a^	Diarrhoea	15	88.2	1	100.0	16	88.9
	Bloody diarrhoea	8	47.1	0	0	8	44.4
	Nausea	6	35.3	1	100.0	7	38.9
	Vomiting	9	53.0	1	100.0	10	55.6
	Abdominal pain	11	64.7	1	100.0	12	66.7
	Fever	7	41.2	1	100.0	8	44.4
	Other symptoms	3	17.6	0	0	3	16.7
Admitted to hospital	Yes	5	29.4	0	0	5	27.8
	No	12	70.6	1	100.0	13	72.2

^a^ Some outbreak cases presented with more than one symptom.

### Analytical study findings

Nine cluster 1 cases and their matched controls were included in the study. Table 2 presents the results of the univariable analysis of food exposures with *p* < 0.2 and OR > 1. Of these, the highest odds of disease due to monophasic *S*. Typhimurium versus other gastrointestinal disease were obtained for Product A of Brand A produced by Company A [mOR = 7.77, 95% CI: 0.89–67.20, *p* = 0.063]. Following forward stepwise selection of variables, no exposures were significant (*p* < 0.05) in the multivariable analysis.

**TABLE 2:** Frequency of consumption of selected food exposures among monophasic *Salmonella* Typhimurium cases (*n* = 9) and controls (*n* = 24), matched case–control study, Ireland, 2022.

	Frequency of exposure						
	Cases (*n* = 9)		Controls (*n* = 24)				
Exposure	*n*	%	*n*	%	Crude mOR	95% CI	*p*‐value
**Chocolate Product A, Brand A, Company A**	7	77.8	4	23.5	7.8	0.9–67.2	0.063
**Chocolate Product B, Brand A, Company A**	2	22.2	1	5.3	5.3	0.5–58.7	0.176
**Any** ^a^ **recalled chocolate Product A–E, Brand A, Company A**	8	88.9	4	23.5	10.5	1.2–88.6	**0.031**
**Any** ^a^ **chocolate Product A–E, Brand B, Company A**	5	83.3	2	10.5	6.8	0.7–62.8	0.089
**Other snack Product A, Brand C, Company B**	5	62.5	6	27.3	4.0	0.7–22.0	0.110
**Other snack Product B, Brand D, Company C**	6	66.7	7	31.8	6.9	0.8–60.5	0.083

Abbreviations: CI, confidence interval; mOR, matched odds ratio.

^a^ Exposure to any product of Brand B or any recalled product of Brand A.

The recall of the implicated products in Ireland included chocolate Product A–E of Brand A produced by Company A. When grouping the recalled products in a composite variable, the odds of disease due to monophasic *S*. Typhimurium versus other gastrointestinal disease were 10.5 times higher for the consumption of at least one of the recalled products [95% CI: 1.24–88.60, *p* = 0.031]. No additional exposures were significant in a multivariable analysis using this composite variable as the start point in a forward stepwise approach with the remaining non‐recalled variables.

### Control measures in Ireland

On 24 March 2022, a national alert was issued to the Departments of Public Health, requesting heightened awareness of salmonellosis among children and urging collection of enhanced surveillance information to ensure that cases were quickly investigated and interviewed. Clinical laboratories were alerted to the need for timely submission of *Salmonella* isolates to the reference laboratory.

On 2 April 2022, the Food Safety Authority of Ireland issued a recall of specific batches of Product A of Brand A produced by Company A. Point‐of‐sale recall notices were displayed in stores supplied with the implicated batches, and a food alert was issued. As the outbreak evolved and with the finding of several positive samples from the production lines of Brand A at the Belgian processing plant, the recall was extended to all batches of products of Brand A by 8 April 2022.

## DISCUSSION

Our investigation identified a monophasic *S*. Typhimurium cluster in Ireland, which formed part of a wider international outbreak. The findings of this Irish analytical study supported the internationally generated hypothesis of a link between illness and specific chocolate products of Brand A. The epidemiological profile of cases in Ireland was consistent with that of cases in the EU/EEA and the UK, predominated by females below the age of 10 years, although a higher proportion of the Irish cases were females in comparison to the international profile (82% versus 63%). Although some cases developed severe clinical symptoms such as bloody diarrhoea, the reported hospitalisation rate in Ireland was lower with 29% versus 40% of all cases hospitalised in other countries [5, 9]. These differences may be explained by differences in health‐seeking behaviour and/or national health service arrangements, although cross‐country differences have not been assessed.

Several outbreaks of salmonellosis have previously been associated with the consumption of chocolate [10, 11, 12]. Chocolate products can function as a particularly effective transmission vehicle. This is related to the long survival time of salmonellae in dry raw material, the protective effect of the high fat content of chocolate in gastric acid and the long shelf‐life of chocolate products. These characteristics have previously facilitated extensive and extended *Salmonella* outbreaks [13, 14]. This outbreak of monophasic *S*. Typhimurium is the second largest ever international outbreak linked to the consumption of chocolate products [10]. As the outbreak evolved, national authorities in the affected countries fed into the international response, coordinated by the ECDC, triggering the publication of a detailed Rapid Outbreak Assessment (ROA) and updates as necessary [4, 5, 15, 16]. Although a two‐month period passed between the initial outbreak identification in the UK and the implementation of control measures in Ireland and the UK on 2 April 2022, closely followed by other countries, the ROA details how country‐specific information on microbiological and traceability investigations pointed toward a Belgian Processing Plant of Company A, which proved essential for the rapid control actions.

The case–control study conducted in Ireland was one of only two analytical epidemiological investigations conducted in the EU/EEA and the UK during this outbreak. Despite international descriptive epidemiological investigations supported by microbiological data, the gathering of analytical epidemiological data provided important confirmatory evidence of the source of the contamination. The small sample size – partly explained by the study timing after product recall – not only posed challenges to the matched analysis due to low numbers and zeros within some discordant pairs but also required the omission of two controls with no matched case questionnaires. This emphasises the importance of undertaking such analytical studies as early into the development of an outbreak as practicable to allow data collection to inform the ongoing investigation, while supporting after action reviews that can improve health system resilience and responsiveness during outbreak investigations.

Salmonellosis is generally under‐reported, and therefore, the outbreak in Ireland and elsewhere is likely to have been larger than the confirmed case count indicates. This is emphasised by the popularity and widespread consumption of the implicated products within the EU/EEA and the UK. The investigation demonstrated the strength of a coordinated response leading to the rapid implementation of control measures. This likely alleviated the impact of the outbreak, particularly as it was developing in the weeks before Easter, during which there are high levels of chocolate purchasing. Following the implementation of control measures, epidemiological and microbiological monitoring continued in Ireland. Except for two cases notified in late May 2022, of which one was a likely secondary case, no additional cases were notified in the weeks following the product recall. Investigations indicated this to be an isolated incident, possibly arising from delayed consumption of the implicated products.

## CONCLUSION

Early identification of the outbreak after the issued EpiPulse alert led to Ireland's rapid involvement in the multicountry collaboration which was crucial to ensure effective and timely information sharing and outbreak control. Rapid action and effective communication were imperative given that chocolate purchasing was at very high levels in the period before Easter. This analytical study provided additional evidence to support collaborative international epidemiological and microbiological findings. Owing to the rapid decline in cases in the weeks following the recall of the implicated products, the control measures were considered to have been effective.

## AUTHOR CONTRIBUTIONS

All authors (Charlotte Salgaard Nielsen, Patricia Garvey, Martin Cormican, Niall DeLappe, Mary Lenahan, Orla Moore, Kay Casey, Suzanne Cotter, Sarah Gee, Benjamin Aniugbo, Gerard Meagher, Elaine Brabazon, Keith Ian Quintyne, Anthony Breslin, RoseMary McBride, Eimear Burke, Margaret White, Martin Cormican, Leah Evans, Catherine Conlon, Annette Dillon, Regina Kiernan, Donna Kilmartin, Cale Lawlor, Ruth McDermott and Paul McKeown) were part of the Outbreak Control Team in Ireland and therefore directly involved in the outbreak investigation, including the gathering of epidemiological and microbiological data which informed the investigation at national level and information sharing at international level. The conceptual and methodological development and analytical investigation were led by the HPSC but enabled by a close collaboration with the Departments of Public Health that contributed to the data collection by conducting the case and control interviews. Charlotte Salgaard Nielsen, Patricia Garvey and Paul McKeown prepared the original and revised draft of the manuscript. All authors reviewed and provided comments to the draft manuscript and approved the final content.

## CONFLICT OF INTEREST STATEMENT

The authors declare no conflicts of interest.

## FUNDING INFORMATION

The authors declare that no funds, grants or other support were received during the preparation of this manuscript.

## Data Availability

The data that support the findings of this study are available upon reasonable request from the corresponding author. The data are not publicly available due to privacy or ethical restrictions.
